# On a Modified Form of Navier-Stokes Equations for Three-Dimensional Flows

**DOI:** 10.1155/2015/692494

**Published:** 2015-03-30

**Authors:** J. Venetis

**Affiliations:** Section of Mechanics, School of Applied Mathematics and Physical Sciences, NTUA, Greece

## Abstract

A rephrased form of Navier-Stokes equations is performed for incompressible, three-dimensional, unsteady flows according to Eulerian formalism for the fluid motion. In particular, we propose a geometrical method for the elimination of the nonlinear terms of these fundamental equations, which are expressed in true vector form, and finally arrive at an equivalent system of three semilinear first order PDEs, which hold for a three-dimensional rectangular Cartesian coordinate system. Next, we present the related variational formulation of these modified equations as well as a general type of weak solutions which mainly concern Sobolev spaces.

## 1. Introduction

The analytical solutions of the initial or boundary value problems of Mathematical Physics, although they cannot be directly inserted into the calculation procedure dealing with conceptual design by engineering viewpoint, still have the advantage compared with the numerical methods of domain discretization and boundary elements that help us to deduce qualitative information (e.g., asymptotic behaviour or stability) for the set of the solutions of the ODE or PDE which describe the evolution of a physical problem [1, 2].

It is well known that Navier-Stokes equations are nonlinear in nature and therefore they are very difficult to be solved analytically. For a detailed study of the nonlinearity of these equations, one may refer to the concepts and results which are performed in excellent monographs by Ladyzhenskaya and Lions [3, 4].

Unfortunately, only a few analytical works are currently present in literature regarding these equations. One of them is the transformation of Navier-Stokes equations to Schrödinger equation by an application of Riccati equation [5]. It has good prospects, since Schrödinger equation is linear and has well-defined solutions. The method of Lie group theory is also applied in order to transform the original partial differential equations into ordinary differential systems [6].

Besides, an analytical solution to three-dimensional incompressible Navier-Stokes equations in Cartesian form has been presented in [7]. The primal idea of this method is that the spatial coordinates are transformed into a single coordinate through a linear transformation. Concurrently, a remarkable contribution has been recently made towards the existence of explicit solutions to these equations [8].

Nevertheless, the existence of weak solutions to the incompressible Navier-Stokes equations in dimension 3 was proved long ago by Leray and Hopf [9, 10]. A comprehensive analysis concerning the uniqueness and regularity of weak solutions, as well as the related questions which are still open, can be found in the literature [9–14].

On the other hand, for the application of Computational Fluid Dynamics (CFD) approaches to unbounded domains (e.g., a half space or the complementary set of a finite space), the contrivance of artificial boundaries is generally demanded. Therefore, one has to suppose primarily that either the shape of the real boundary or the rates of a field quantity along the artificial boundary are considered as known beforehand. Besides, the creation of a finite element grid constitutes a difficult geometrical problem, perhaps more difficult as it was proved than the circumstantial physical problem that a CFD method is called to solve.

The key point here is that a mesh must be predefined to provide a certain relationship between the nodes, which is the base of the formulation of these conventional numerical methods [15].

However, it is known from modern Algebra [16] that every partition of a Cartesian product can be motivated univocally by a binary relation of equivalence, that is, reflexive, symmetric, and transitive, which subdivides the circumstantial domain into mutually disjoint subdomains, whose union is the original set. Under this viewpoint, one can also consider the creation of a finite element grid as a partition of a Cartesian product and evidently the mesh points are the common vertices (*x*_*n*_, *y*_*n*_, *z*_*n*_) of the finite elements, which for convex polyhedral domains in *R* ^3^ are mainly trihedral or tetrahedral elements, such that they form a partition of the corresponding domain. Hence, every ordered triple in the form (*x*_*n*_, *y*_*n*_, *z*_*n*_) constitutes a mesh point or a node of a finite element grid if and only if it satisfies a binary relation *σ* of equivalence; that is, *σ*(*x*_*n*_, *y*_*n*_, *z*_*n*_) = 0 for all *n* ∈ *N*.

In fact, every numerical solution by means of domain discretization methods is the approximation of the functional values at the mesh points, which evidently are discrete points.

However, we have to elucidate that if one does not prove the independency of the numerical solution for any possible distribution of the mesh points, then the spatial coordinates will be functionally dependent on each other something opposite with Eulerian formalism.

In other words, the resulting numerical solution should not depend on the selection of the binary relation of equivalence which relates the discrete variables *x*_*n*_, *y*_*n*_, *z*_*n*_ to one another.

To overcome this difficulty, new discretization methods have been introduced such as mesh-free methods and domain-free discretization method. The main concept for mesh-free methods is to establish a system of algebraic equations for the whole problem domain without the use of a predefined mesh. Mesh-free methods use a set of nodes scattered within the problem domain as well as sets of nodes scattered on the boundaries of the domain to represent (not discretize) the problem domain and its boundaries. These sets of scattered nodes do not form a mesh, which means that no information on the relationship between the nodes is required, at least for field variable interpolation [15].

Meanwhile, domain-free discretization method directly solves partial differential equations in the standard coordinate system. It can be easily applied to solve irregular domain problems without introducing the coordinate transformation technique. The primal idea of this method is inspired by the analytical method. Consequently, the differential equation and its discrete form are irrelevant to solution domain. Therefore, the discrete form of PDEs can involve some points outside the solution domain or may not be the mesh points. The functional values at these points can be calculated by finding the approximate form of the solution [17].

## 2. Investigation of Incompressible Viscous Flow Patterns

In a Cartesian frame of reference, the fundamental equations of mass and momentum conservation for the generic case of an unsteady, incompressible, and viscous flow field are represented in vector terms in a domain *Ω* × (0, *T*] ⊂ *R* ^4^ as follows:

(1)  ∇·V→=0

(2)  ∂V→∂t+V→·∇V→=−1ρ∇P+g→+μρ∇2V→.

The term $\left(\vec{V}\cdot \nabla \right)\vec{V}$ is actually a pseudovector expression and its expansion in other than Cartesian coordinates is rather inconvenient. Therefore, if one expresses this acceleration term in true vector form the following equivalent system arises:

(1) ∇·V→=0

(3)  ∂V→∂t+∇V22−V→×∇×V→=−1ρ∇P+g→+μρ∇2V→.

This system is completed by the following conditions:

(4) $$\begin{array}{c} \vec{V}\left(x,y,z,0\right)={\vec{V}}_{0}\;\text{in}\;\Omega , \end{array}$$

(5) $$\begin{array}{c} \vec{V}\left(x,y,z,t\right)=\vec{0}\;\mathrm{on}\;\partial \Omega \times \left(0,T\right]. \end{array}$$

Meanwhile, it is known from Vector Calculus that the following identity holds [18]:

(6) $$\begin{array}{c} \nabla \left(\nabla \cdot \vec{V}\right)\equiv \nabla \times \left(\nabla \times \vec{V}\right)+\nabla^{2}\vec{V}. \end{array}$$

Hence (3) can be written out in the following form:

(7) ∂V→∂t+∇V22−V→×∇×V→ =−1ρ∇P+g→+μρ∇(∇·V→)−μρ∇×∇×V→.

Since $\nabla \cdot \vec{V}=0$, it follows

(8) ∂V→∂t+∇V22−V→×∇×V→ =−1ρ∇P+g→−μρ∇×∇×V→.

Let $d\vec{s}$ be an element of length, which evidently is not infinitesimal, along an arbitrary streamline of the flow field.

In the sequel, let us operate at both members of (8) the dot product with $d\vec{s}$ to obtain

(9) ∂V→∂t·ds→+∇V22·ds→−V→×∇×V→·ds→ =−1ρ∇P·ds→+g→·ds→−μρ∇×∇×V→·ds→.

Since $d\vec{s}$ is by definition collinear to $\vec{V}$, the vector $\vec{V}\times \left(\nabla \times \vec{V}\right)$ is perpendicular to $d\vec{s}$, and therefore

(10) $$\begin{array}{c} \vec{V}\times \left(\nabla \times \vec{V}\right)\cdot d\vec{s}=0. \end{array}$$

Hence, (9) yields

(11) ∂V→∂t·ds→+∇V22·ds→ =−1ρ∇P·ds→+g→·ds→−μρ∇×∇×V→·ds→.

According to the commutative property of the dot product, the term $\nabla \times \left(\nabla \times \vec{V}\right)\cdot d\vec{s}$ can be equivalently represented as follows [18]:

(12) $$\begin{array}{c} d\vec{s}\cdot \nabla \times \left(\nabla \times \vec{V}\right)=\left(d\vec{s}\times \nabla \right)\cdot \left(\nabla \times \vec{V}\right). \end{array}$$

Besides, let $\vec{r}=(x,y,z)$ be the respective position vector, which is obviously drawn from the origin to an arbitrary point of this aforementioned arbitrary streamline of the flow field. Hence, we can consider the following vector:

(13) $$\begin{array}{c} \Delta \vec{r}=\left(x_{i},y_{i},z_{i}\right)-\left(x_{j},y_{j},z_{j}\right), \end{array}$$

where the points (*x*_*i*_, *y*_*i*_, *z*_*i*_) and (*x*_*j*_, *y*_*j*_, *z*_*j*_) are the circumstantial endpoints of the element of length $d\vec{s}$.

Obviously, the vector $\Delta \vec{r}$ is the chord of the corresponding arc defined by the vector $d\vec{s}$.

Letting the rate of the arc *ds* become infinitesimal, it implies that

(14) $$\begin{array}{c} d\vec{s}=d\vec{r}. \end{array}$$

On the other hand, let us also introduce two continuous vector functions

(15a) ξ→:R4⟶R3:ξ→=i→ξ1x,y,z,t+j→ξ2x,y,z,tξ→:R4⟶R3:ξ→ +k→ξ3(x,y,z,t)

and

(15b) ζ→:R4⟶R3:ζ→=i→ζ1x,y,z,t+j→ζ2x,y,z,tζ→:R4⟶R3:ζ→  +k→ζ3(x,y,z,t)

such that

(16a) $$\begin{array}{c} \vec{V}=\vec{\xi }\times \vec{\zeta } \end{array}$$

or equivalently

(16b) i→ζ3ξ2−ζ2ξ3−j→ζ3ξ1−ζ1ξ3+k→ζ2ξ1−ζ1ξ2 ≡i→Vx+j→Vy+k→Vz.

Here, we can suppose without violating the generality that both vectors $\vec{\xi }$, $\vec{\zeta }$ are not fixed; that is, the derivatives of their components with respect to spatial coordinates are never zero. Hence the determinants of the related coefficient matrix either for $\vec{\xi }$ or for $\vec{\zeta }$ cannot be identically zero; therefore (16b) can be solved univocally with respect to $\vec{\xi }$ or $\vec{\zeta }$ in terms of $\vec{V}$.

Concurrently, it is also known [19] that every solenoid vector field can be represented as the cross product of the gradients of two scalar multivalued functions, such as to be sufficiently differentiable with respect to their independent variables.

Hence the following statement holds:

(17a) $$\begin{array}{c} \vec{\xi }=\nabla f_{1} \end{array}$$

(17b) ∧ζ→=∇f2,

where *f*_1_ : *R* ^4^ → *R* and *f*_2_ : *R* ^4^ → *R*.

Moreover, let us also make the additional assumption that each time rate of the vector $\vec{\zeta }$ can be always represented as the vector difference ${\vec{r}}_{2}-{\vec{r}}_{1}$ of two position vectors ${\vec{r}}_{1}$, ${\vec{r}}_{2}$ which are obviously drawn from the origin to the endpoints of $\vec{\zeta }$.

It is known from Vector Calculus that provided two arbitrary position vectors ${\vec{r}}_{1},{\vec{r}}_{2}\in R^{3}$ the following statement holds:

(18) $$\begin{array}{c} \vec{r}=\left(1-a\right){\vec{r}}_{1}+a{\vec{r}}_{2},\;\forall a\in \left(0,1\right], \end{array}$$

where the endpoint of position vector $\vec{r}$ must belong to the segment which is defined by the vector ${\vec{r}}_{2}-{\vec{r}}_{1}$.

Subsequently, the following claim can be formulated:

(19) $$\begin{array}{c} \forall t\in R\;\;\exists a\in \left(0,1\right]:\vec{\zeta }\left(x,y,z,t\right)=\frac{\vec{r}-{\vec{r}}_{1}}{a}. \end{array}$$

Actually, this claim does not violate the generality of the mathematical formalism that is motivated by (16a), because it is known that the interval (0,1] has the power of the Continuum; that is, it has the same cardinality with the set *R*∖{0}.

Besides, the curl of velocity can be modified as follows:

(20) ∇×V→=∇×ξ→×ζ→⟺∇×V→=∇×ξ→×r→−r→1a⟺∇×V→=1a∇×ξ→×r→−1a∇×ξ→×r→1.

In addition, it is known from Vector Calculus that for any constant vector $\overset{⃑}{Α}\in R^{3}$ the following two identities hold [19]:

(21a) $$\begin{array}{c} \nabla \times \left(\overset{⃑}{Α}\times \vec{r}\right)\equiv 2\overset{⃑}{Α}, \end{array}$$

(21b) $$\begin{array}{c} \left(\vec{r}\times \overset{⃑}{Α}\right)\equiv \frac{1}{2}\nabla \times \left(\left(\vec{r}\cdot \vec{r}\right)\overset{⃑}{Α}\right), \end{array}$$

where $\vec{r}=(x,y,z)$.

Here, one may consider that these statements can be also applicable for each time rate of the vector function $\vec{\xi }$.

Thus, (20) by the use of (21a) yields

(22a) $$\begin{array}{c} \nabla \times \vec{V}=\frac{2}{a}\vec{\xi }-\frac{1}{a}\nabla \times \left(\vec{\xi }\times {\vec{r}}_{1}\right), \end{array}$$

where ${\vec{r}}_{1}=(x_{1},y_{1},z_{1})$.

Also, according to identity (21b) we deduce that

(22b) ξ→×r→1=−r→1×ξ→⟺(ξ→×r→1)=−x12+y12+z122∇×ξ→.

Thus, (20) with the aid of (22b) becomes

(23) $$\begin{array}{c} \nabla \times \vec{V}=\frac{2}{a}\vec{\xi }+\frac{{x_{1}}^{2}+{y_{1}}^{2}+{z_{1}}^{2}}{2a}\nabla \times \left(\nabla \times \vec{\xi }\right). \end{array}$$

Next, (23) can be combined with (17a) and (22a) to show that

(24) ξ→×r→1=−x12+y12+z122∇×∇×∇f1⟺ξ→×r→1=0→.

Finally, by modifying the right member of (12) taking into account (22a) and (24) one obtains

(25) ds→×∇·∇×V→=ds→×∇·2aξ→⟺ds→×∇·∇×V→=2ads→·∇×ξ→⟺ds→×∇·∇×V→=2a∇×ξ→·ds→=0→⟺(ds→×∇)·(∇×V→)=0→.

Here, (17a) has also been taken into consideration.

Then, we can perform (11) in the following equivalent modified form:

(26a) ∂V→∂t·ds→+∇V22·ds→=−1ρ∇P·ds→+g→·ds→⟺∂V→∂t+∇V22+1ρ∇P−g→⊥ds→

(26b) ∨∂V→∂t+∇V22=−1ρ∇P+g→.

We have to clarify that the above disjunction holds only along the streamline network of the flow patterns which we examine here.

Also, we can remark that streamlines indicate local flow direction, not speed, which usually varies along a streamline.

In continuing, let us focus on relationship (26a). According to Eulerian formalism of motion, the spatial coordinates are functionally independent of each other, as well as on the variable of time. Therefore, the vector term $\partial \vec{V}/\partial t$ must be collinear to the vector $\vec{V}$. In addition, since the type of the flow fields that we investigate is incompressible and also the gravitational field is obviously conservative, the sum of the rest terms on the left member of relationship (26a) can be written in Cartesian coordinates as follows:

(26c) ∇V22+1ρ∇P−g→ =∇V22+1ρPx,y,z,t−Ux,y,z,

where the gravitational potential *U*(*x*, *y*, *z*) is generally described by the formula

(26d) $$\begin{array}{c} U\left(x,y,z\right)=\overset{}{\underset{\Omega }{∭}}\frac{G\rho }{r\left(x,y,z\right)}\;dx\;dy\;dz. \end{array}$$

Hence, relationship (26a) yields

(26e) $$\begin{array}{c} \frac{\partial \vec{V}}{\partial t}+\nabla \left(\frac{V^{2}}{2}+\frac{1}{\rho }P\left(x,y,z,t\right)-U\left(x,y,z\right)\right)⊥d\vec{s}. \end{array}$$

Since $\partial \vec{V}/\partial t//d\vec{s}$, we can infer that the statement (26e) cannot hold.

Thus, the initial system of the governing equations (1) and (2) or equivalently (1) and (3) substantially reduces to (26b).

We can also pinpoint that in (26b) does not occur any term containing the dynamic viscosity of the fluid; hence this equation can hold for either inviscid or viscous incompressible flows as well as for any type of shear and free turbulence.

To apply this equation to the distribution of velocity field of a particular flow, it is necessary to define boundary conditions on solid surfaces if they are present and at infinity if a uniform stream is involved. This uniform stream usually constitutes a rigid lid for the flow, through which only molecular mass transfer can be carried out [20, 21]. Therefore, the kinematic condition which concerns the equality of the normal components of velocity at the boundary and evidently arises from purely kinematical considerations when there is no mass transfer across the boundary implies that velocity component which is perpendicular to the free stream surface must vanish; that is,

(27a) $$\begin{array}{c} \vec{V}\cdot {\vec{n}}_{1}=\vec{0}, \end{array}$$

where ${\vec{n}}_{1}$ is the unit normal to the free stream surface.

One exception, when mass transfer across the free stream surface takes place, concerns two-dimensional Prandtl's boundary layer flows [22]. In addition, since free stream velocity is generally motivated by a steady flow, it follows that streamlines, streaklines, and pathlines are coincident [20, 21]. This also implies that along the free stream surface the following condition holds:

(27b) $$\begin{array}{c} \frac{\partial \vec{V}}{\partial t}=\vec{0}. \end{array}$$

For frictionless flows, since the viscous stresses are identically zero, the velocity gradients can be infinite and therefore the no-slip condition along a solid boundary does not hold. Hence, the boundary condition which remains is the kinematic one which implies that

(27c) $$\begin{array}{c} \vec{V}\cdot {\vec{n}}_{2}=\vec{0}. \end{array}$$

Here, ${\vec{n}}_{2}$ is the unit normal to the solid boundary.

For viscous flows, the no-slip and kinematic boundary conditions on a solid surface require that the fluid adjacent to the surface has the velocity of the surface which in a motionless Cartesian frame of reference is zero.

However, speaking for the no-slip condition we should elucidate that there are some exceptions concerning some types of laminar and turbulent flows [23–26], where this condition does not hold.

Besides, since no flow occurs across a streamline, therefore a streamline meets the same stipulation as a solid boundary and also close to a boundary wall the flow direction must be parallel to the boundary; one can consider, without violating the nature of the investigated phenomenon, the solid boundary as a stream surface of the investigated flow.

This conclusion arises from the fact that in any three-dimensional unsteady flow field the instantaneous streamlines are the orbits of the vector $\vec{V}(x,y,z,t)=\left(V_{x}(x,y,z,t),V_{y}(x,y,z,t),V_{z}(x,y,z,t)\right)$ at time *t*.

Hence, since according to (5) $\vec{V}(x,y,z,t)=\vec{0}$ for all (*x*, *y*, *z*, *t*)∈∂*Ω* × (0, *T*] the above statement is warranted.

On the other hand, it is known that for a given instant of time a stream surface of a flow field, which, roughly speaking, is defined as a bundle of neighbouring streamlines, is created by a streamline which moves along a plane curve remaining parallel to a given line. This curve is the directrix of the stream surface and the stream line is the generator. Obviously, the directrix curve is not unique because any plane intersects a given stream surface along a plane curve that can serve as directrix.

However, since there is only one streamline passing through any particular velocity point of a flow field, but infinite stream surfaces that can contain it, the notion of the principal stream surface is more appropriate to describe the solid boundary of a flow field [27]. The principal stream surface is defined as a stream surface whose tangent planes are the rectifying planes of the streamlines within it. The normal of each point on this surface has the same direction as the streamline's principal normal vector, and its magnitude is defined to be the magnitude of velocity at that point.

This definition extends to the case of the solid boundary of a flow field for all rates of the variable of time on the interval (0, *T*], since the shape of the solid boundary evidently stays invariant.

Furthermore, ((27a), (27b)) guarantee that the above reasonable assumption may also concern the free stream surface.

Consequently, whenever the no-slip and kinematic conditions for a solid boundary hold, (5) which complete (1), (3) can also complete (26b). In addition, ((27a), (27b)) must be also taken into account if a uniform stream is involved.

Meanwhile, we should mention that sometimes it is convenient to express a vector equation such as (26b) in forms other than the Cartesian ones, for example, to describe viscous flows past cylinders or spheres. The two other three-dimensional frames which are commonly applied are the cylindrical polar coordinate and the spherical polar coordinate. These frames extend the polar coordinate system to three dimensions and along with Cartesian one are known as orthogonal coordinate systems [28].

Nevertheless, in situations where an injective mapping is necessary between Cartesian and cylindrical or spherical polar coordinates, one should primarily exclude the *zz*′ since to each ordered triple (0,0, *z*) ∈ *R* ^3^ there corresponds an indefinite ordered triple in the form (0, *θ*, *z*) for all *θ* ∈ [0,2*π*) in a cylindrical polar frame and also the indefinite ordered triples in the forms (*r*, *θ*, 0) or (*r*, *θ*, *π*) for all *θ* ∈ [0,2*π*) in a spherical polar frame of reference. Consequently, in a cylindrical polar system, to each point (*x*, *y*, *z*) ∈ *R* ^3^ with the exception of the axis *zz*′ there corresponds a definite ordered triple (*r*, *θ*, *z*) such that *r* ≥ 0, 0 ≤ *θ* < 2*π*, −*∞* < *z* < +*∞*, with the exception of the axis *zz*′. Also, in a spherical polar system, to each point (*x*, *y*, *z*) ∈ *R* ^3^ with the exception of the axis *zz*′ there corresponds a definite ordered triple (*r*, *θ*, *ϕ*) such that *r* ≥ 0, 0 ≤ *θ* < 2*π*, 0 ≤ *ϕ* ≤ *π*. However, even with these restrictions, a problem that still remains is the following.

The equation of a curve in cylindrical or spherical polar coordinates may be satisfied by points whose ordered triples are not in accordance with the above restrictions. This may happen because such an equation is generally a binary relation of the variables *r*, *θ*, *z* or *r*, *θ*, *ϕ* and its domain of definition is not necessarily a subset of the above defined sets. Also, to localize the vertical and horizontal asymptotes to a curve in cylindrical and spherical polar coordinates is generally a difficult procedure.

According to the above data and given that (26b) holds on streamlines network of an incompressible flow, the application of this relation in other than Cartesian coordinates is preferable for external flows past convex geometric solids whose boundaries encircle the axis *zz*′ or for flow fields described by closed streamlines.

On the other hand, (26b) can be further applied to more specific cases of viscous incompressible flows where some terms of (2) can be neglected beforehand. For instance, one may refer to low Reynolds number flows where evidently the viscous forces are dominant.

These flows are divided into the following two major categories.

(i) When the inertial forces are not neglected: this implies that viscous forces have the same order of magnitude as inertial and pressure gradient forces [20] and therefore (26b) holds as is.
(ii) When the inertial forces are a priori neglected (e.g., fully developed duct flows, flows through porous media, groundwater movement, etc.): then (2) results in the creeping motion equation:
  (28) $$\begin{array}{c} \frac{\partial \vec{V}}{\partial t}=-\frac{1}{\rho }\nabla P+\vec{g}+\frac{\mu }{\rho }\nabla^{2}\vec{V}. \end{array}$$

Evidently, for steady state flows the latter reduces to Stokes equation.

In addition, as is known from the literature [20], in creeping flows the pressure satisfies Laplace equation that is

(29) $$\begin{array}{c} \nabla^{2}P=0. \end{array}$$

According to (1) and (6), (28) becomes

(30) $$\begin{array}{c} \frac{\partial \vec{V}}{\partial t}=-\frac{1}{\rho }\nabla P+\vec{g}-\frac{\mu }{\rho }\nabla \times \left(\nabla \times \vec{V}\right). \end{array}$$

Hence, the method that we have already proposed can be applied to yield

(31) $$\begin{array}{c} \frac{\partial \vec{V}}{\partial t}=-\frac{1}{\rho }\nabla P+\vec{g}. \end{array}$$

Here, we should denote that the latter relationship holds only on streamline network of the flow.

A qualitative property which automatically emerges is that, along any streamline of such a flow field, the sum $\partial \vec{V}/\partial t+(1/\rho )\nabla P$ is time-independent, since it varies only with the elevation above an arbitrary datum.

Suggestively, let us examine an open channel creeping flow in a rectangular parallelepiped (length *a*, width *b*, and height *c*), such that *a* ≫ *b* and *a* ≫ *c*. The main characteristic of this flow is that the upper boundary is freely deformable, in contrast to the solid boundaries. The boundary conditions at the free surface are always that both the pressure and the shear stress vanish everywhere. Apparently, (31) includes the latter one of these aforementioned conditions, since it does not contain the viscous terms.

Also, the pressure at the free surface coincides with atmospheric pressure which is conventionally taken to be zero.

Hence, the Dirichlet conditions which complete (29) are formulated as follows:

(32a) $$\begin{array}{c} P\left(x,y,c\right)=0; \end{array}$$

(32b) $$\begin{array}{c} P\left(x,y,0\right)=f\left(x,y\right). \end{array}$$

We will seek the solution of (29) by the method of separation of variables [29, 30] placing

(33) $$\begin{array}{c} P\left(x,y,z\right)=X\left(x\right)\cdot Y\left(y\right)\cdot Z\left(z\right). \end{array}$$

Thus, the application of this well-known method to the above Dirichlet problem finally yields

(34) $$\begin{array}{c} P=\overset{\infty }{\underset{m=1}{\sum }}\overset{\infty }{\underset{n=1}{\sum }}A_{mn}\sin\left(\frac{m\pi x}{a}\right)\sin\left(\frac{m\pi y}{b}\right)\sinh\left[k_{mn}\left(z-c\right)\right], \end{array}$$

where the coefficients *A*_*mn*_ must be determined by incorporating the homogeneous condition.

Consequently, by inserting (34) into (30) and integrating both sides with respect to *t* one obtains an explicit representation of velocity for this flow pattern.

In continuing, let us return to (26b) and represent it in the following form:

(35) $$\begin{array}{c} \frac{\partial \vec{V}}{\partial t}+\mathrm{grad}\left(\frac{{\vec{V}}^{2}}{2}+\frac{1}{\rho }P\left(x,y,z,t\right)-U\left(x,y,z\right)\right)=\vec{0}. \end{array}$$

The generic type of (35) can be also expressed in terms of their algebraic rates of all vector quantities for each axis of a motionless Cartesian coordinate system as follows:

(36a) $$\begin{array}{c} \frac{\partial V_{x}}{\partial t}+\frac{\partial F_{1}\left(V_{x}\right)}{\partial x}=0, \end{array}$$

(36b) $$\begin{array}{c} \frac{\partial V_{y}}{\partial t}+\frac{\partial F_{2}\left(V_{y}\right)}{\partial y}=0, \end{array}$$

(36c) $$\begin{array}{c} \frac{\partial V_{z}}{\partial t}+\frac{\partial F_{3}\left(V_{z}\right)}{\partial z}=0, \end{array}$$

where

(37a) ∂F1Vx∂x=Vx∂Vx∂x+12·∂Vy2+Vz2∂x +∂∂x1ρPx(x,y,z,t)−U(x,y,z),

(37b) ∂F2Vy∂y=Vy∂Vy∂y+12·∂Vx2+Vz2∂y +∂∂y1ρPy(x,y,z,t)−U(x,y,z),

(37c) ∂F3Vz∂z=Vz∂Vz∂z+12·∂Vx2+Vy2∂z +∂∂z1ρPzx,y,z,t−Ux,y,z.

We emphasize again that we have arrived at the above equations, taking into account that the Eulerian formalism for fluid motion asserts us that all velocity components are functionally independent of each other.

This basic assumption is actually in accordance with the validity of the general principle of separation of motion for each axis of a Cartesian coordinate system, which holds either for individual particles or for Continuum media.

Let us also remark that any multivalued function *u* with domain of definition of the set *R* × [0, +*∞*), such that its support supp*u* is to be a compact subset of *R* × [0, +*∞*), is referred to as test function.

In the case of bounded domains a set is referred to as compact if and only if it is closed and bounded. Hence, the conceptual supposition that supp*u* is a compact subset of *R* × [0, +*∞*) implies that there exists a compact set in the form [*a*, *b*]×[0, *T*] ⊂ *R* × [0, +*∞*) such that supp*u* ⊂ [*a*, *b*]×[0, *T*].

Therefore, to derive the variational forms of the semilinear differential equations ((36a), (36b), and (36c)) we can multiply them, respectively, with the arbitrary test functions *u*_1_(*x*, *t*), *u*_2_(*y*, *t*), and *u*_3_(*z*, *t*) and next integrate with respect to variables *x* and *t*, *y* and *t*, and *z* and *t* provided that supp*u*_*i*_ ⊂ [*a*_*i*_, *b*_*i*_]×[0, *T*_*i*_], *i* = 1,2, 3.

Hence, we deduce

(38a) ∫0+∞∫−∞+∞∂Vx∂t+∂∂xF1Vxu1dx dt =∫0T1∫a1b1∂Vx∂t+∂∂xF1Vxu1dx dt=0,

(38b) ∫0+∞∫−∞+∞∂Vy∂t+∂∂yF2Vyu2dy dt =∫0T2∫a2b2∂Vy∂t+∂∂yF2Vyu2dy dt=0,

(38c) ∫0+∞∫−∞+∞∂Vz∂t+∂∂zF3Vzu3dz dt =∫0T3∫a3b3∂Vz∂t+∂∂zF3Vzu3dz dt=0.

Meanwhile, according to the definition of test function, the following equalities also arise:

(39a) $$\begin{array}{c} u_{1}\left(x,T_{1}\right)=0\;\forall x\in R, \end{array}$$

(39b) $$\begin{array}{c} u_{2}\left(y,T_{2}\right)=0\;\forall y\in R, \end{array}$$

(39c) $$\begin{array}{c} u_{3}\left(z,T_{3}\right)=0\;\forall z\in R. \end{array}$$

Besides, let us also place

(40a) $$\begin{array}{c} V_{x}\left(x,y,z,0\right)=\phi_{1}\left(x,y,z\right), \end{array}$$

(40b) $$\begin{array}{c} V_{y}\left(x,y,z,0\right)=\phi_{2}\left(x,y,z\right), \end{array}$$

(40c) $$\begin{array}{c} V_{z}\left(x,y,z,0\right)=\phi_{3}\left(x,y,z\right). \end{array}$$

In order for the group of (39a), (39b), and (39c) to be in accordance with initial condition (4) which concerns the three components of $\vec{V}$ one can assume without violating the generality that *T*_1_ = *T*_2_ = *T*_3_ = *T*.

Suggestively, let us focus on (38a). Obviously, this process is exactly the same for ((38b), (38c)).

Thus, we place

(41a)  I1=∫0T1∫a1b1∂∂xF1Vxu1dx dt,

(41b)  I2=∫0T1∫a1b1∂Vx∂tu1dx dt.

Hence, it is evident that

(42) $$\begin{array}{c} I_{1}+I_{2}=0. \end{array}$$

We can manipulate each one integral separately. Thus, we can write out

(43) I1=∫0T1∫a1b1∂∂xF1Vxu1dx dt =∫0T1∫a1b1∂∂xF1Vxu1−F1Vx∂u1∂xdx dt⟺I1=∫0T1∫a1b1∂∂xF1Vxu1dxdt⟺I1 −∫0T1∫a1b1F1Vx∂u1∂x dx dt⟺I1=∫0T1F1Vxb1,tu1b1,t      −F1Vxa1,tu1a1,tdt⟺I1 −∫0T1∫a1b1F1Vx∂u1∂x dx dt⟺I1=−∫0T1∫a1b1F1(Vx)∂u1∂x dx dt

or equivalently

(44) $$\begin{array}{c} I_{1}=-\overset{+\infty }{\underset{0}{\int }}\overset{+\infty }{\underset{-\infty }{\int }}F_{1}\left(V_{x}\right)\frac{\partial u_{1}}{\partial x}\;dx\;dt. \end{array}$$

On the other hand, the second term of (42) is expanded as follows:

(45) I2=∫0T1∫a1b1∂Vx∂tu1dx dtI2=∫0T1∫a1b1∂∂tVxu1−Vx∂u1∂tdx dt⟺I2=∫0T1∫a1b1∂∂tVxu1dx dt−∫0T1∫a1b1Vx∂u1∂t dx dt⟺I2=∫a1b1∫0T1∂∂tVxu1dt dx−∫a1b1∫0T1Vx∂u1∂t dt dx⟺I2=∫a1b1∫0T1∂∂tVxu1dtdx−∫a1b1∫0T1Vx∂u1∂t dt dx.

Taking into consideration (39a) and (40a), we can point out that

(46) ∫0T1∂∂tVxu1dt =Vxx,y,z,T1u1x,T1−Vxx,y,z,0u1x,0⟺∫0T1∂∂tVxu1dt=−Vxx,y,z,0u1x,0⟺∫0T1∂∂tVxu1dti=−ϕ1x,y,zu1x,0.

Hence, we deduce

(47) $$\begin{array}{c} I_{2}=-\overset{b_{1}}{\underset{a_{1}}{\int }}\overset{T_{1}}{\underset{0}{\int }}V_{x}\frac{\partial u_{1}}{\partial t}\;dt\;dx-\overset{b_{1}}{\underset{a_{1}}{\int }}\phi_{1}\left(x,y,z\right)u_{1}\left(x,0\right)dx \end{array}$$

or equivalently

(48) I2=−∫−∞+∞∫0+∞Vx∂u1∂t dt dx −∫−∞+∞ϕ1x,y,zu1x,0dx.

According to the above data, the classical solution of (36a) for every test function *u*_1_ : *R* ^2^ → *R* must satisfy the following relationship:

(49) ∫−∞+∞∫0+∞F1Vx∂u1∂x+Vx∂u1∂tdt dx +∫−∞+∞ϕ1x,y,zu1x,0dx=0.

Apparently, when the function *V*_*x*_ : *R* ^4^ → *R* is successively integrable with respect to *x* and *t* over every Cartesian product in the form [*a*, *b*]×(0, +*∞*) and also satisfies (49) for every test function *u*_1_ : *R* ^2^ → *R*, it is referred to as a weak solution of (36a) with the initial condition (39a).

However, a shortcoming of (49) is that noncontinuous functions are also admitted as solutions of (36a) with the initial condition (39a), something opposite with Continuum Mechanics standpoint [31].

Nevertheless, the derivatives here are generalized derivatives and the choice of spaces is made to ensure the existence of all integrals. Therefore, every classical solution of (36a) with the initial condition (39a) satisfies the variational equation (49).

## 3. Discussion

As it was previously stated, (26b) constitutes an equivalent representation of equations of mass and momentum conservation for incompressible flows, which have been expressed in true vector form. The main difference between these relationships and the proposed one is that in (26b) the viscous terms do not occur, and also this formula holds on the streamline network. Hence, a firm conclusion that can be drawn is that, along any streamline of such a flow, the viscosity does not contribute to velocity rate of change. Moreover, the integration of this equation between any two arbitrary points of the flow field is only possible if the flow behaves locally as inviscid and also the body forces are conservative, except perhaps some singular points as in the potential vortex. Actually, Navier-Stokes equations may be used with a high degree of accuracy for physical problems involving viscosity variations if the viscosity gradient is not too large. Besides, according to the constitutive relation for a Newtonian fluid it is known that momentum is transferred through the fluid by the viscous action and therefore the kinematic viscosity is also referred to as the momentum diffusivity of the fluid, that is, the ability of the fluid to transport momentum, in the perpendicular direction of the flow. Although this concept was based on unidirectional flows, it is also valid in three dimensions. Hence, since the streamlines indicate local flow direction, the explicit expressions of velocity components in terms of spatial coordinates and time which may arise from (26b) have already incorporated the local velocity changes caused by the interaction amongst adjacent fluid regions.

On the other hand, a variational formulation of (26b) in Cartesian coordinates was also presented, something that enables us to obtain weak solutions in Sobolev spaces. On the understanding that in these spaces the notion of the weak derivative plays the central role, in order to examine when a weak solution in the sense of (49) also constitutes a classical solution, it is necessary to know further properties of Sobolev spaces [32]. In particular one needs to investigate the following.

- What classical differentiability properties does the weak solution fulfil?
- In what sense does the weak solution satisfy the circumstantial boundary conditions?

In the event that the above information has been received, one can approximate functions in Sobolev spaces by functions that are differentiable in the classical sense. Hence, various useful properties of Sobolev spaces can be proved by first verifying them for classical functions and then extending them to Sobolev spaces.

Moreover, another item that has not been thoroughly investigated so far is whether a weak solution can develop singularities in finite time, even considering smooth initial data [10, 14].

## 4. Conclusions

The objective of this paper was to propose a method to eliminate the nonlinear terms of Navier-Stokes equations for three-dimensional, incompressible viscous flows, resulting in a modified form which holds along the streamline network.

The main characteristic of this recasted form is that it does not contain the viscous terms, and therefore this equivalent representation can hold either for frictionless or for viscous flows as well as for any type of shear or free turbulence.

A variational formulation of this relationship was also presented; hence one may obtain weak solutions in Sobolev spaces.

In closing, we can also remark that a weak formulation of this original problem provides us with related methods (e.g., Galerkin method) for approximating its circumstantial weak solution in a properly chosen finite-dimensional subspace of functions with good approximation properties that are also suitable for numerical computations.
